# Educación interprofesional y práctica colaborativa: Estrategias para transformar un sistema de salud

**DOI:** 10.15446/rsap.V26n3.114943

**Published:** 2024-05-01

**Authors:** Arturo José Parada-Baños

**Affiliations:** 1 AP: MD. Esp. Ginecología y Obstetricia. M. Sc. Educación. Universidad Nacional de Colombia. Bogotá, Colombia. ajparadab@unal.edu.co Universidad Nacional de Colombia Universidad Nacional de Colombia Bogotá Colombia ajparadab@unal.edu.co

**Keywords:** Educación interprofesional, competencia profesional, sistema de salud, ciencias de la salud, equidad en salud, calidad de la atención de salud *(fuente: DeCS, BIREME)*, Interprofessional education, professional competence, health systems, health sciences, health equity, quality of health Care *(source: MeSH, NLM)*

## Abstract

En un mundo donde la atención en salud es cada vez más compleja y fragmentada, la colaboración efectiva entre diferentes profesionales de la salud se vuelve esencial. La educación interprofesional y la práctica colaborativa (EIPC) emergen como enfoques innovadores que tienen el potencial de transformar los sistemas de salud y reevaluar los modelos de educación para la formación del talento humano en salud. Este ensayo examina la necesidad de la EIPC en la formación de profesionales de la salud y su impacto en la mejora de los sistemas de salud y explora la profundidad y el alcance de esta estrategia pedagógica, argumentando que su implementación puede mejorar significativamente la calidad y la equidad de la atención en salud, y la satisfacción de los usuarios y de los profesionales. Sin embargo, la implementación de la EIPC enfrenta desafíos significativos, incluyendo la falta de apoyo institucional, recursos limitados y barreras culturales y profesionales. El artículo concluye que, a pesar de estos desafíos, la EIPC tiene el potencial de transformar positivamente los sistemas de salud y la educación en ciencias de la salud, promoviendo una atención más colaborativa e integrada.

## Abstract (en)

In a world where health care is increasingly complex and fragmented, effective collabo-ration between different health professionals becomes essential. Interprofessional education and collaborative practice (EIPC) emerge as innovative approaches that have the potential to transform health systems and reevaluate education models for the training of human talent in health. This essay examines the need for EIPC in the training of health professionals and its impact on the improvement of health systems and explores the depth and scope of this pedagogical strategy, arguing that its implementation can significantly improve the quality and equity of health care, and the satisfaction of users and professionals. However, the implementation of EIPC faces significant challenges, including lack of institutional support, limited resources, and cultural and professional barriers. The article concludes that, despite these challenges, EIPC has the potential to positively transform health systems and health sciences education, promoting more collaborative and integrated care.

En la actualidad, los sistemas de salud enfrentan dinámicas cambiantes que requieren adaptaciones constantes, impulsadas por la transición demográfica y epidemiológica, enfermedades infecciosas nuevas, emergentes y reemergentes como la COVID-19 y la integración de tecnologías avanzadas en la atención, el diagnóstico y el tratamiento. Estos cambios exigen una atención integral, integrada, de calidad, humanizada y resolutiva que ponga a la persona, a la familia y a la comunidad en el centro del modelo de atención.

Sin embargo, la educación en ciencias de la salud sigue estando predominantemente anclada en un modelo centrado en la formación uniprofesional, lo cual limita la capacidad de los futuros profesionales para enfrentar las demandas actuales de atención en salud.

La educación interprofesional y práctica colaborativa (EIPC) ha resurgido en la última década, ya no solo como una estrategia pedagógica en el campo de la educación en ciencias de la salud y en la atención primaria, como se ha desarrollado en países como el Reino Unido, Estados Unidos y Canadá 1-3, sino como una estrategia mundial para la transformación de los modelos de atención en los sistemas de salud. Su potencial para mejorar la equidad en la atención, la calidad de los servicios, optimizar el uso de los recursos, apoyar las metas de cobertura universal y la distribución del talento humano, junto con el fortalecimiento del trabajo en equipos interprofesionales, el liderazgo colaborativo y el reconocimiento de roles, tiene un impacto significativo en la calidad del cuidado, la seguridad del paciente y la satisfacción profesional.

La EIPC representa una ruptura en el marco congelado, por más de un siglo, de la educación superior para la formación del talento humano en salud, que sigue los preceptos flexnerianos 4 de la medicina con una formación fragmentada entre lo básico y lo clínico, creando subjetividades profesionales centradas en la salud como enfermedad. Esto se refleja en modelos de atención centrados en la enfermedad y la curación, perpetuando concepciones políticas, económicas y sociales en los sistemas de salud de muchos países de América Latina, empobrecidos por la fragmentación y la segmentación que llevan a sistemas ineficaces e inequitativos.

Tres aspectos fundamentales han sido transversales en estos modelos de atención: la salud como enfermedad, la hegemonía médica en la toma de decisiones y la salud como poder político para el dominio social. La EIPC surge como una "estrategia del futuro" que busca una alianza entre la educación y la salud para deconstruir y reconstruir una nueva concepción cultural que muestre a la salud como bienestar, desmitificando.

Esto implica repensar la educación superior para la formación del talento humano en salud y el desarrollo del ejercicio profesional en todos los niveles de atención. Es en este punto donde la EIPC puede generar un punto de inflexión para transformar la estructura misma de racionalidad política, social y económica de un modelo de atención en salud.

## Contexto y necesidades de la educación interprofesional y práctica colaborativa

La dinámica de los modelos de atención dentro de un sistema de salud conlleva ajustes y cambios constantes, impulsados por la transición demográfica y epidemiológica 5, la aparición de nuevas enfermedades, emergentes y el avance de nuevas tecnologías y biotecnologías en el diagnóstico y en el tratamiento. Además, las estrategias tecnológicas para abordar la atención, el impacto de la cultura y el uso de redes sociales por parte de los usuarios, la promoción y protección de los derechos humanos, los nuevos modelos económicos y de innovación, y las diversas estrategias de atención que ponen a la persona, la familia y la comunidad en el centro son factores clave en la sostenibilidad y efectividad del sistema 6. Todo esto se orienta a ofrecer una atención integral, de calidad, humanizada y resolutiva.

Contrariamente, la educación en ciencias de la salud para la formación del talento humano avanza a un ritmo más pausado. Muchas facultades de medicina aún conservan modelos del siglo pasado sin grandes modificaciones. Esto resulta en una formación uniprofesional que, aunque sólida en conocimientos específicos, presenta limitaciones para responder a las dinámicas actuales de la atención en salud y la formación del talento humano. En la actualidad, se promueve la comunicación interdisciplinaria e intercultural, el reconocimiento de roles profesionales, la práctica colaborativa efectiva, el trabajo en equipo, el liderazgo compartido y la toma conjunta de decisiones, todas dinámicas propias del enfoque interprofesional centrado en la persona, la familia y la comunidad 7.

Ante esta necesidad de adaptación y modernización, la Organización Mundial de la Salud (OMS) estableció en 2010 el "Marco de referencia sobre Educación Inter-profesional y Práctica Colaborativa", instando a todos los países a incluir la EIPC en los programas de formación de profesionales en ciencias de la salud y sociales, tanto de pregrado como de posgrado. El objetivo es mejorar la calidad de la educación y formar talento humano capacitado en la práctica colaborativa y la conformación de equipos interprofesionales para la atención en salud 8.

La EIPC resurge no solo como una estrategia pedagógica, donde estudiantes de dos o más profesiones aprenden juntos para mejorar la colaboración y la calidad de la atención en salud 1, sino también como un enfoque estratégico para el ejercicio profesional. Este enfoque tiene el potencial de transformar positivamente los sistemas de salud globales.

En el ámbito de las políticas de salud, la EIPC se ha convertido en una herramienta fundamental para alcanzar tres de los Objetivos de Desarrollo Sostenible: el objetivo 3 (salud y bienestar), promoviendo la equidad en la atención y la salud para todos; el objetivo 4 (educación de calidad), armonizando las políticas de educación con las de salud; y el objetivo 8 (trabajo decente y crecimiento económico), promoviendo condiciones adecuadas de bienestar para los profesionales de la salud en áreas rurales y dispersas.

La EIPC, al fomentar la práctica colaborativa, impacta positivamente en la economía de los países y en la cobertura universal en salud, favoreciendo el desarrollo de prácticas integradas que garantizan la equidad. Los equipos interprofesionales son más propensos a garantizar la cobertura en territorios rurales y rurales dispersos, lo que mejora el acceso y la oportunidad de atención. La OMS/OPS han subrayado que la EIPC es crucial para alcanzar la quíntuple meta de mejora de la salud mundial.

En 2007, el Institute for Healthcare Improvement (IHI) desarrolló el modelo de la triple meta en salud: mejorar la experiencia del paciente, mejorar la salud de las poblaciones y reducir el costo per cápita de la atención médica. Este marco se convirtió en una guía para los principales sistemas de salud. En 2014, se añadió una cuarta meta: mejorar la satisfacción del personal de salud. Durante la pandemia de COVID-19 en 2021, se incluyó una quinta meta: lograr la equidad en la atención de salud a nivel global 9.

## Desafíos y barreras para la implementación efectiva de la EIPC

La relevancia de la EIPC se respalda con evidencia científica que demuestra su utilidad en la formación del talento humano en salud. La EIPC ha mostrado mejoras en habilidades de comunicación y trabajo en equipo, comprensión de roles y responsabilidades de otras disciplinas, liderazgo y trabajo colaborativo, así como en el desarrollo de habilidades de resolución de problemas y toma de decisiones interprofesionales, y la optimización del uso de recursos físicos y tecnológicos 10-12.

De igual manera, ha demostrado beneficios significativos en los procesos de atención en salud. Estos incluyen la atención integral y de calidad, el incremento de la seguridad del paciente, el fomento de actitudes laborales positivas en el trabajo en equipo, la promoción de la salud y el bienestar del paciente, la familia y la comunidad, y el favorecimiento de la práctica colaborativa centrada en el paciente 13-15.

Además, la EIPC ha mostrado resultados positivos en diversos entornos y poblaciones, entre los cuales se encuentran programas de formación en salud 1-3, odontología 16, profesionales de salud, escuelas de medicina 17, práctica profesional y sistemas de salud 18, fisioterapia 19, enfermería 20, adultos mayores 21, obstetricia y ginecología 22, pediatría 23 y otros en múltiples áreas y disciplinas de la salud.

**Cuadro 1:**
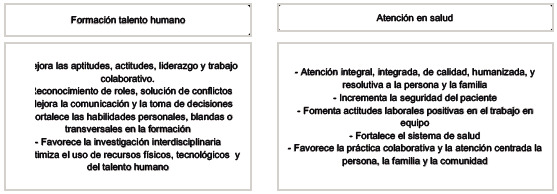
Beneficios de la EIPC en la formación del talento humano y la atención en salud

Sin embargo, a pesar de la creciente evidencia científica, de los compromisos de los gobiernos con los entes mundiales y de los escenarios políticos favorables, muchos programas de ciencias de la salud siguen siendo indiferentes y reacios a implementar e institucionalizar la EIPC 24.

La implementación de la EIPC enfrenta numerosos desafíos y debilidades que afectan su sostenibilidad 25. Estos desafíos incluyen la falta de apoyo institucional, limitaciones de recursos financieros y disponibilidad de talento humano 26. También se identifican problemas como el poco interés de docentes y estudiantes, ya sea por desconocimiento, falta de interés o distribución de tiempos 27, y problemas de gestión y coordinación entre programas y asignaturas relacionados con el currículo propio de cada programa. Además, la cultura profesional establecida desde las instituciones educativas y arraigada en la atención en salud presenta un obstáculo significativo, ya que implica modificar estructuras y prácticas culturales propias de cada disciplina profesional 28.

**Cuadro 2:**
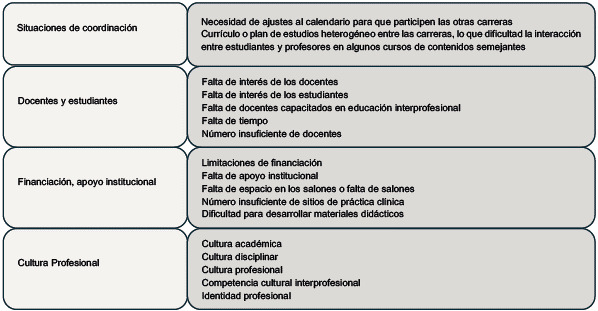
Barreras en la implementación de la EIPC

## Cultura profesional y sus desafíos en la implementación de la EIPC

Como se mencionó anteriormente, la atención en salud se ve influida por factores que generan un arraigo en modelos centrados en la enfermedad. A nivel de la educación superior, uno de los mayores obstáculos para la apertura hacia una educación centrada en el estudiante y el trabajo interprofesional es la cultura profesional 29. Esta cultura, entendida como el conjunto de valores, creencias y normas que rigen la práctica de cada disciplina, desempeña un papel fundamental en la formación y el ejercicio del talento humano en salud. Sin embargo, ha sido uno de los factores menos estudiados al evaluar las dificultades y las barreras para la implementación y la sostenibilidad de la educación interprofesional en los programas de ciencias de la salud.

De acuerdo con Rovere 30, las diferentes profesiones de la salud han evolucionado bajo sus propias fuerzas históricas y las influencias sociológicas de la sociedad. Cada profesión ha trabajado para definir su identidad, sus valores, sus espacios de práctica, su rol en la atención al paciente y su legitimidad social. Por esta razón, hablar de EIPC resulta desafiante, cuando no amenazante, en muchos escenarios.

La formación uniprofesional, que se ha desarrollado a lo largo de la historia de las disciplinas en las ciencias de la salud, genera un marco sociocultural de pensamiento centrado en el profesional 29. Esto hace que cada profesión tenga, frente a su objeto disciplinar, su propia competencia cultural 31,32. Esta situación deja como consecuencia la ausencia de lógicas profesionales de encuentro, la separación de roles y la delimitación de funciones en clave de territorios profesionales que no se pueden invadir.

Una de las razones detrás de este fenómeno es la socialización profesional. Cada disciplina de la salud tiene su propio proceso de formación y socialización, lo que lleva a los profesionales a desarrollar una identidad y perspectiva disciplinaria específica. Esto puede generar una mentalidad centrada en su propia disciplina y una tendencia a mantener límites rígidos entre las profesiones, lo que dificulta la colaboración interprofesional.

El centrismo profesional se refiere a la tendencia de cada disciplina o profesión a centrarse en su propio conocimiento, perspectivas y prácticas, relegando o minimizando la importancia de otras disciplinas o profesiones. Esta mentalidad centrada en la profesión genera relaciones de poder asimétricas entre las disciplinas y profesiones de la salud, tensiones relacionadas con el poder, desigualdades de género, equidad, jerarquías y autoridad. Estas tensiones influyen en la toma de decisiones, la distribución de los recursos, las oportunidades de desarrollo profesional y las dinámicas de liderazgo en los entornos de salud.

Las relaciones entre profesionales se basan en interconsultas disciplinarias y apoyos multidisciplinarios para la toma de decisiones, lo que implica la asunción distante de responsabilidades unilaterales en el manejo de un usuario. Sin embargo, hay pocos modelos que promuevan la toma de decisiones integrales de un equipo de trabajo interprofesional con responsabilidades individuales pero liderazgo colaborativo, lo cual mejoraría la calidad de la atención y la optimización en el uso de los recursos.

El mayor desafío en una visión transformadora de la educación para la formación del talento humano en salud está en el reconocimiento de las coordenadas socioculturales de cada profesión y en la enseñanza de una competencia cultural interprofesional. Esta competencia se entiende como la capacidad de los profesionales para comprender y adaptarse a las diferencias culturales disciplinares y trabajar de manera efectiva en equipos interprofesionales diversos.

## Desafíos de la EIPC en la educación superior y los sistemas de salud en Latinoamérica

La implementación de la Educación Interprofesional y la Práctica Colaborativa (EIPC) enfrenta desafíos significativos tanto en los sistemas de salud de Latinoamérica como en la educación superior en ciencias de la salud.

Para que la educación en la formación del talento humano sea efectiva, debe alinearse con un sistema de salud que aspire a brindar una atención integral, integrada, de calidad, humanizada y resolutiva. Esto incluye incluye, entre otros, la promoción de la salud, la prevención de las enfermedades y la atención a las necesidades de bienestar de las personas, familias y comunidades. En este contexto, la educación debe fomentar el desarrollo integral de las capacidades de los estudiantes, abarcando habilidades cognitivas, procedimentales, actitudinales, emocionales, sociales y éticas. Estas habilidades deben promover la flexibilidad, la adaptabilidad, la colaboración y el trabajo en equipo, creando una cultura de práctica colaborativa interprofesional entre las diversas disciplinas para implementar un enfoque de atención centrado en la persona, la familia y la comunidad.

El modelo de educación por capacidades difiere del enfoque por competencias y resultados de aprendizaje medibles que hoy predomina en la educación superior. Mientras que el enfoque por competencias tiende a fomentar la competitividad, el individualismo y las jerarquías, el modelo por capacidades busca desarrollar habilidades más complejas y adaptativas, necesarias en un entorno de atención integral y humanizada. Este modelo debe ser más compatible con un enfoque holístico y centrado en la persona.

Para la implementación efectiva de la EIPC, es fundamental armonizar la formación académica del talento humano con las necesidades del sistema de atención en salud. No se puede esperar que una formación tradicional, realizada en silos disciplinarios, produzca de repente verdaderos equipos interprofesionales capaces de ofrecer atención integral en áreas urbanas, rurales o rurales dispersas.

La EIPC puede adaptarse a diversos sistemas de salud, como se ha observado en el Reino Unido, donde la atención primaria en salud es la puerta de entrada al sistema nacional de salud 33. Un enfoque similar se encuentra en el Sistema Único de Salud de Brasil 34, y en Canadá, donde el sistema nacional de salud combina la prestación directa de servicios por el Estado con sistemas de aseguramiento financiados parcialmente por el contribuyente 35. En Canadá, la entrada al sistema también se realiza a través de la atención primaria en salud. Australia, con su sistema de salud mixto, que incluye seguro médico universal financiado por el Estado, seguro de salud privado y servicios de salud pública, también prioriza la atención primaria a través de los médicos generales 36.

En estos países, la EIPC tiene una gran fortaleza y es una parte integral de sus sistemas de salud. Un denominador común en todos estos sistemas es la importancia central de la atención primaria en salud y la formación de equipos interprofesionales. Además, en estos países, la EIPC es fundamental en la educación superior en ciencias de la salud, siendo incluso un criterio de acreditación de alta calidad en varios de ellos 37,38.

La EIPC representa más que una simple estrategia pedagógica, es una herramienta poderosa que tiene el potencial de transformar los sistemas de salud desde sus cimientos. Su implementación efectiva puede impactar positivamente en la calidad y la equidad de la atención, el bienestar de los usuarios y el desarrollo profesional del talento humano en salud. A pesar de los desafíos significativos que presenta su adopción, es crucial para aquellos que buscan mejoras estructurales en los sistemas de salud, en los modelos de atención y en los enfoques educativos.

La EIPC y la práctica colaborativa tienen el potencial de revolucionar los sistemas de salud al mejorar la calidad del cuidado, la seguridad del paciente y la satisfacción profesional. Estos enfoques son especialmente relevantes cuando se comparan con los modelos de salud tradicionales, que a menudo se centran en la enfermedad desmitificando la concepción de la salud como ausencia de enfermedad. La EIPC, por su parte, promueve un enfoque más holístico y equitativo, centrado en la persona, la familia y la comunidad, y es más congruente con un modelo de atención integral y humanizado.

Para que la EIPC sea efectiva, es necesario un enfoque integral que aborde las barreras culturales, educativas e institucionales. Esto implica desarrollar políticas de apoyo, promover la formación continua y fomentar un cambio cultural que valore la colaboración interprofesional. Es esencial que las instituciones educativas adapten sus currículos para incluirla y que los sistemas de salud adopten prácticas que faciliten el trabajo en equipos interprofesionales.

La implementación de la EIPC también requiere superar desafíos como la resistencia al cambio, la falta de recursos y la necesidad de coordinación entre disciplinas. Sin embargo, los beneficios superan con creces estos obstáculos. Al promover una cultura de práctica colaborativa interprofesional, no solo se mejora la calidad de la atención, sino que se optimiza el uso de los recursos y aumenta la satisfacción de los profesionales de la salud.

A manera de conclusión, se puede decir que EIPC es una estrategia transformadora que merece ser tenida en cuenta en cualquier esfuerzo por mejorar los sistemas de salud y la educación en ciencias de la salud. Su adopción puede llevar a una atención más integral, equitativa y de alta calidad, beneficiando tanto a los pacientes como a los profesionales. Para lograr estos objetivos, es necesario un compromiso conjunto de las instituciones educativas, los sistemas de salud y los profesionales, trabajando unidos hacia un futuro más colaborativo y centrado en el bienestar de todos ♦
